# *Streptococcus pneumoniae* as a Cause of Community-Acquired Pneumonia in Indian Adolescents and Adults: A Systematic Review and Meta-Analysis

**DOI:** 10.1177/1179548419862790

**Published:** 2019-07-31

**Authors:** Canna J. Ghia, Raja Dhar, Parvaiz A Koul, Gautam Rambhad, Mark A Fletcher

**Affiliations:** 1 Medical and Scientific Affairs, Pfizer Limited, Mumbai, India; 2 Consultant Pulmonologist, Department of Pulmonology, Fortis Hospital, Kolkata, India; 3 Department of Internal and Pulmonary Medicine, Sher-i-Kashmir Institute of Medical Sciences, Srinagar, India; 4 Emerging Markets Medical, Pfizer Inc, Paris, France

**Keywords:** Community-acquired pneumonia, Streptococcus pneumoniae, bacterial pneumonia, pneumonia, aetiology

## Abstract

**Background:**

*Streptococcus pneumoniae* is one of the primary cause of community-acquired pneumonia (CAP) worldwide. However, scant data are available on the prevalence of etiological organisms for CAP in adolescent and adult Indian population.

**Objective:**

We performed a systematic review and meta-analysis to determine the contribution of *S. pneumoniae* in the causation of CAP in Indian patients aged 12 years or above.

**Methodology:**

We performed a systematic search of both indexed and non-indexed publications using PubMed, databases of National Institute of Science Communication and Information Resources (NISCAIR), Annotated Bibliography of Indian Medicine (ABIM), Google Scholar, and hand search including cross-references using key terms ‘community acquired pneumonia AND India’. All studies, published between January 1990 and January 2017, that evaluated Indian patients aged above 12 years with a confirmed diagnosis of CAP were eligible for inclusion. Our search retrieved a total of 182 studies, of which only 17 and 12 qualified for inclusion in the systematic review of all etiological organisms, and meta-analysis of *S. pneumonia*, respectively.

**Results:**

A total of 1435 patients met the inclusion criteria. The pooled proportion of patients with *S. pneumoniae* infection was 19% (95% confidence interval [CI]: 12%-26%; I^2^ = 94.5% where I^2^ represents heterogeneity, *P* < .01). Other major etiological agents are *Mycoplasma pneumoniae* (15.5% [1.1%-35.5%]), *Klebsiella pneumoniae* (10.5% [1.6%-24.0%]), and *Legionella pneumophila* (7.3% [2.5%-23.8%]).

**Conclusions:**

Analysis found approximately a one-fifth proportion of adult Indian patients of CAP with *S. pneumoniae* infection, suggesting it as a leading organism for causing CAP compared with other etiological organisms.

## Introduction

*Pneumococcal pneumonia* comprises about two-thirds of all bacterial pneumonia and is the most common cause of morbidity in patients with community-acquired pneumonia (CAP).^1^ A recent global burden of disease report estimated that there were 291.8 million episodes of lower respiratory tract infection (LRTI) (95% uncertainty interval [UI] 276.3 million to 307.0 million) each year. More than one-third of these episodes (101.8 million) occurred in children less than 5 years of age.^2^ In a 2017 global burden of disease study, *Streptococcus pneumoniae* was the most commonly identified LRTI pathogen in all age groups, causing more than 1.5 million LRTI deaths. In particular, there were 0.7 deaths in patients above 70 years and 0.4 million fatalities among children less than 5 years of age. In India, CAP due to *S. pneumoniae* was responsible for 82 000 deaths among children less than 5 years of age.^2^

Empirical therapy for CAP starts with antibiotics, which include those that target *S. pneumoniae*; however, the misuse of antibiotics can lead to drug resistance. Consequently, empirical microbial treatment for CAP should be based on the knowledge of the causative pathogen to avoid treatment failure and the associated costs. Studies reveal a case-fatality rate ranging from 4% to 33% where there was an incorrect initial selection of antibiotics.^3-5^

There is some Indian literature on the microbiological aetiology of CAP among adults.^6^ Hence, this systematic review and meta-analysis were performed to analyse the proportion of CAP due to *S. pneumoniae* infection in Indian patients >12 years of age.

## Methodology

This systematic review and meta-analysis were conducted in accordance with the Preferred Recording Items for Systematic Reviews and Meta-Analysis (PRISMA).

### Eligibility criteria for studies

All studies, published between January 1990 and January 2017, that evaluated Indian patients aged above 12 years of age with a confirmed diagnosis of CAP were eligible for inclusion. Adolescence was taken to begin at 12 years of age.

### Exclusion criteria for studies

All studies on CAP patients that were conducted outside India or/and not conducted in Indian population were excluded from the analysis. Studies were also excluded if they were conducted in Indian patient populations <12 years of age or where the full text was not available.

### Measurements

The primary outcome of this study was the proportion of patients with CAP caused by *S. pneumoniae*. The secondary outcome was to determine the proportion of all other aetiological agents causing CAP. Sensitivity analysis was carried out based on the reporting quality of the included studies.

### Search strategy

We performed a systematic search on PubMed, using the key terms ‘Community-Acquired Pneumonia AND India’, ‘Community-Acquired Pneumonia AND aetiology’, ‘Community-Acquired Pneumonia AND Diagnosis’, or ‘Community-Acquired Pneumonia AND Management’. The search was performed after applying constant filters based on these additional search criteria: Article Types – Randomized Clinical Trials, Meta-Analysis, Systematic Literature Reviews, Literature Reviews, Observational Studies; Language – English; Publication Date – 01/01/1990-08/01/2017; Species – Humans; Adult and Adolescent – 12+ years. Additional records were identified through other sources (the National Institute of Science Communication and Information Resources [NISCAIR], the Annotated Bibliography of Indian Medicine [ABIM], and Google Scholar) using the search terms: ‘Community-Acquired Pneumonia AND ‘India’. A hand search was also performed using the same key terms, based on cross-references and review of journals from the library. A medical librarian was not involved in designing or reviewing the research strategy.

### Risk of bias

The risk of bias was avoided by assessing the quality of information from each study. The instrument used to assess the risk of bias analysis was the instrument developed by Joanna Briggs Institute for systematic reviews addressing questions of prevalence.^7^

### Data extraction

Data was collected from all the primary studies using a structured sheet in Microsoft Excel. Any discrepancies arising while entering the data were sorted out by discussion among all the contributors. Two reviewers were involved in determining the risk of bias analysis and data extraction. Reviewers resolved any disagreements by discussion between themselves. Study characteristics extracted included authors details, year of publication, title of study, place of study, and type of study. Patient parameters included number of study participants and their mean age, gender, educational level, and marital status. CAP was classified by aetiology.

### Statistical analysis

A meta-analysis of proportion for aetiological agents with corresponding 95% confidence interval (CI) for all included individual studies was performed. Also, meta-analysis using a random effects method (DerSimonian and Laird), with the assumption of a degree of heterogeneity (*i*)^2^ among the studies, was performed. The outcomes were presented as pooled estimates with 95% CI.^8^ The *i*^2^ test assessed variation in the outcome of all included studies with respect to the primary and secondary objectives. The meta-analyses were carried out using open software.^9^

## Results

**Figure 1.**
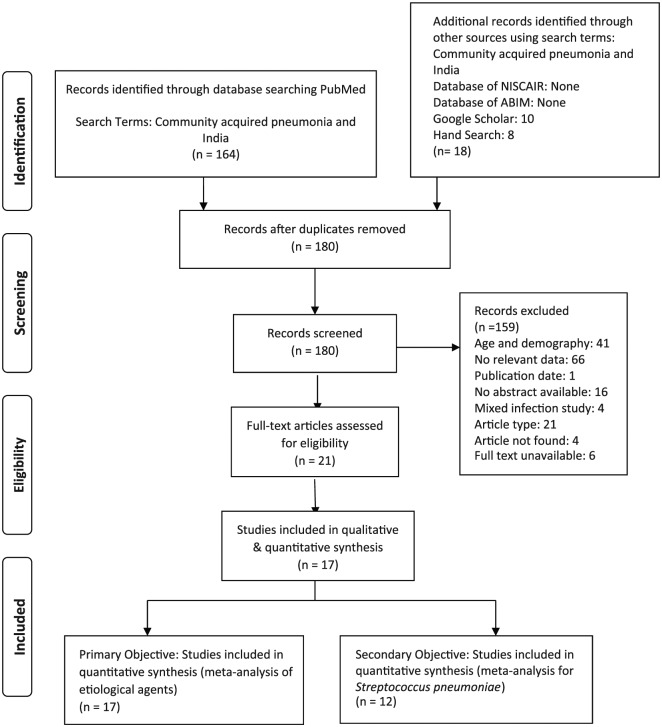
PRISMA flow diagram. ABIM indicates Annotated Bibliography of Indian Medicine; NISCAIR, National Institute of Science Communication and Information Resources.

PubMed searches retrieved 164 studies and Google Scholar searches retrieved ten. Eight additional studies were retrieved via hand search. The ABIM and NISCAIR database searches did not retrieve any relevant study. The analysis identified 17 relevant studies (Figure 1). All 17 studies^10-26^ were considered for qualitative as well as the quantitative synthesis of aetiological agents. Ultimately, only 12 of 17 studies were included for *S. pneumoniae* meta-analysis,^10,13,15,16,18-22,24-26^ since the remaining five studies did not include *S. pneumoniae* among the aetiological agents in their analyses. Table 1 represents the characteristics of the studies included in the analysis.

**Table 1.** Study characteristics table.

First author	Year of publication	Study design	Number of patients	Risk factors/aetiology	Diagnostic test	Mean age (years) (full range) of population	Geographical location/type of hospital
Shah et al	2010	Prospective study	150	*Streptococcus pneumonia, Klebsiella pneumonia, Mycoplasma pneumonia*	Sputum gram stain and culture, blood culture	53.68 years (15-80)	Northern India (Srinagar)/Tertiary Care Hospital
Dharmadhikari et al	2013	Observational study	104	*S. pneumoniae, K. pneumoniae, Staphylococcus aureus, Pseudomonas aeruginosa*	Sputum gram stain and culture, blood culture	Not available	Pune (India)/Tertiary Care Hospital
Mythri et al	2013	Cross-sectional observational clinical study	100	*S. pneumoniae, Klebsiella* spp., *Pseudomonas* spp.	Sputum gram stain and culture, blood culture	Not available	Bangalore (India)/Tertiary Care Hospital
Sreekanth and Reddy	2015	Prospective observational study	50	*S. pneumoniae, K. pneumoniae, Enterobacter, Escherichia coli, P. aeruginosa, S. aureus*	Bronchoscopy, sputum analysis and culture	Mean age not available (Minimum age, 18 years, but maximum age, not available)	Andhra Pradesh (India)/Tertiary Care Hospital
Bansal et al	2004	Prospective observational study	70	*S. pneumoniae, K. pneumoniae, S. aureus, M. pneumoniae, E. coli*, beta-haemolytic streptococci, other Gram-negative bacilli	Blood culture, sputum stain and culture, pleural fluid culture, ELISA	52.77 years (17-93)	Himachal Pradesh/Tertiary Care Hospital
Dey et al	1997	Prospective observational study	72	*S. pneumoniae, K. pneumoniae*	Blood culture, sputum stain and culture	50.6 years (18-80)	New Delhi/Tertiary Care Hospital
Dey et al	2000	Prospective observational study	62	*M. pneumoniae*	Blood culture, sputum analysis and culture, gelatin particle agglutination test, ELISA	41.77 years (14-67)	New Delhi/Tertiary Care Hospital
Khadanga et al	2014	Observational study	Enrolled: 464; culture isolated: 149	*S. pneumoniae, K. pneumoniae, P. aeruginosa, S. aureus*	Blood culture, sputum stain and culture	Not available	Madhya Pradesh and Odisha/Tertiary Care Hospital
Ravindranath et al	2016	Observational study	150	*S. aureus*	Blood Culture, sputum stain and culture	55.71 years (<40 years and ⩾80)	Hyderabad/Tertiary Care Hospital
Jain et al	2014	Prospective observational study	120	*S. pneumoniae, K. pneumoniae, S. aureus, Haemophilus influenza*, other Gram-negative bacilli	Sputum analysis, blood culture	52.36 years (15-85)	Gwalior/Tertiary Care Hospital
Kejriwal et al	2015	Prospective observational study	60	*S. pneumoniae, K. pneumoniae, P. aeruginosa, E. coli, Acinetobacter, S. aureus*	Sputum stain and culture, ELISA	Mean age not available (Minimum age, 14 years, but maximum age, not available)	Mumbai/Tertiary Care Hospital
Shrikhande et al	2015	Prospective observational study	50	*S. pneumoniae, K. pneumoniae, S. aureus, P. aeruginosa, E. coli*	Sputum stain and culture, blood culture, pleural fluid	49.34 years (Minimum age, 12 years, but maximum age, not available)	Rajasthan/Tertiary Care Hospital
Acharya et al	2014	Prospective observational study	100	*S. pneumoniae, K. pneumoniae, P. aeruginosa*	Sputum stain and culture	Mean age not available (14-70)	Mangalore, Coastal Karnataka/Tertiary Care Hospital
Menon et al	2013	Prospective observational study	145	*K. pneumoniae, P. aeruginosa, alpha-haemolytic streptococci, E. coli, beta-haemolytic streptococci, Atypical coli*	Sputum culture	Mean age not available (18-90)	Kerala/Secondary Care Hospital
Anbumani et al	2010	Observational study	470 samples	*Legionella pneumophila*, other aetiological agents of bacterial pneumonia	Sputum stain and culture, blood culture, pleural fluid analysis, endotracheal aspirate	Mean age not available (24-76)	Tirupati/Tertiary Care Hospital
Angrup et al	2016	Observational study	134	*L. pneumophila*	Blood culture, urine analysis, respiratory tract fluids (throat swab, nasopharyngeal aspirates, endotracheal aspirates, bronchoalveolar lavage, sputum culture), PCR, ELISA	The study included paediatric and adult patients, but age not mentioned	New Delhi/Tertiary Care Hospital
Dorairaj et al	2015	Observational study	107	*M. pneumoniae, Chlamydia pneumoniae*	Sputum culture, urine antigen, serological diagnosis by ELISA	44.42 years (18 to >65)	Chennai/Tertiary Care Hospital

Abbreviations: ELISA enzyme-linked immunosorbent assay; PCR, polymerase chain reaction.

### Risk of bias analysis

The risk of bias assessment was delineated in the reporting of the following items (Supplementary Table 1): adequate sample size, appropriate recruitment, appropriate reporting of study subjects and of setting, reliable and objective measurement of outcome condition, statistical analysis, and accountability for confounding factors.

### Primary outcome

The meta-analysis included 1435 patients. The patients’ ages ranged from 12 to 93 years, with a predominance of the male gender. Clinical diagnosis was made both by physical examination along with a chest X-ray. The microbiological assessment of sputum culture was made in all studies, while in 12 studies, both sputum and blood cultures were obtained (Table 1).

**Figure 2.**
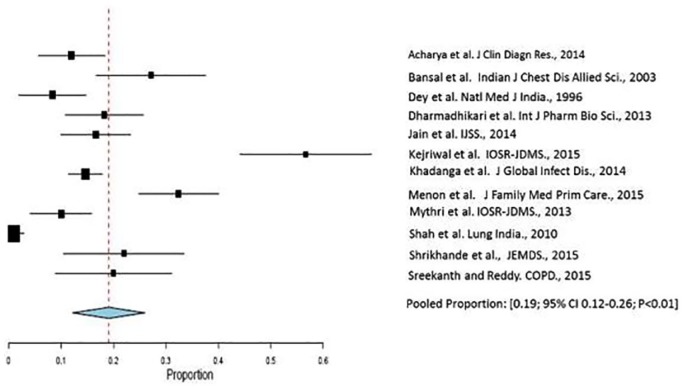
Forest plot displaying meta-analysis of proportion of *Streptococcus pneumoniae* infection in patients with community-acquired pneumonia. Binary random effects model was applied to get pooled proportion and 95% confidence interval (0.19; 95% CI 0.12–0.26; P < .01).

The pooled proportion of patients with *S. pneumoniae* infection was 19% (95% CI: 12%-26%; I^2^ = 94.5%; *P* < .01) (Tables 2 and 3, Figure 2). The degree of heterogeneity was significant.^10,13,15,16,18-22,24-26^

**Table 2.** Proportion of *Streptococcus pneumoniae* infection in patients with community-acquired pneumonia.

Study Reference	Proportion of prevalence of *S. pneumoniae*	CI lower	CI upper	Weight%
Acharya et al^10^	0.12	0.05	0.19	8.68
Bansal et al^13^	0.27	0.15	0.39	7.73
Dey et al^15^	0.08	0.02	0.15	8.68
Dharmadhikari et al^16^	0.18	0.10	0.26	8.46
Jain et al^18^	0.17	0.09	0.24	8.62
Kejriwal et al^19^	0.57	0.38	0.76	7.16
Khadanga et al^20^	0.15	0.11	0.18	9.18
Menon et al^21^	0.32	0.23	0.42	8.41
Mythri and Nataraju^22^	0.10	0.04	0.16	8.78
Shah et al^24^	0.01	−0.01	0.03	9.30
Sreekanth and Reddy^26^	0.20	0.08	0.32	7.45
Shrikhande et al^25^	0.22	0.09	0.35	7.55

Abbreviation: CAP, community-acquired pneumonia; CI, confidence interval.

**Table 3.** Pooled aetiology data of studies.

Etiological agent (EA)	No. of subjects with EA	Total no. of subjects with confirmed culture report	Total no. of subjects with CAP	Pooled proportion (%)	Median (IQR)
*S. pneumoniae*	257	699	1435	19.0	17.5 (11.7)
*K. pneumonia*	151	699	1435	10.52	9.8 (13.1)
*M. pneumoniae*	37	128	239	15.48	6.5 (17.2)
*P. aeruginosa*	89	699	1435	6.20	6.3 (4.5)
*S. aureus*	79	570	1468	5.38	7.0 (7.2)
*Acinetobacter*	14	330	604	2.31	2.9 (2.3)
*Enterobacter*	5	176	340	1.47	1.2 (1.1)
*E. coli*	38	539	899	4.22	4.5 (2.8)
*L. pneumophila*	44	604	604	7.28	13.2 (10.7)

Abbreviations: CAP, community-acquired pneumonia; IQR: interquartile range.

### Secondary outcome

**Figure 3.**
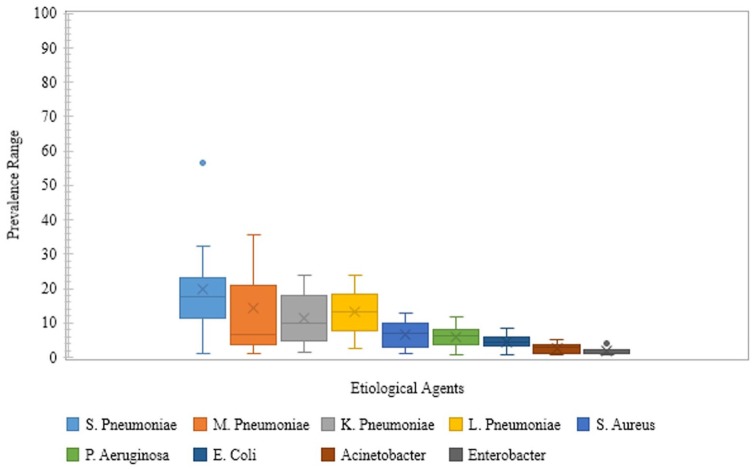
Prevalence range of etiological agents in community-acquired pneumonia in Indian setting. Blue and Grey dots are outliers; the cross (X) mark depicts the Mean.

Other causative organisms of CAP in the Indian adolescent and adult population were *Mycoplasma pneumoniae* (1.1%-35.4%),^13,14,17^ *Klebsiella pneumoniae* (1.6%-24.0%),^10,13,15,16,18-22,24-26^ *Legionella pneumophila* (2.5%-23.8%),^11,12^ *Staphylococcus aureus* (1.0%-12.8%),^10,13,15,16,18-22,24-26^ *Pseudomonas aeruginosa* (0.83%-11.6%),^10,13,15,16,18-22,24-26^ *Escherichia coli* (0.83%-8.57%),^10,13,16,18,19,21,22,24-26^ *Acinetobacter* spp. (0.83%-5.0%),^10,13,16,18,19,24,26^ and *Enterobacter* spp. (0.83%-4.0%)^10,13,18,26^ (Supplementary Table 2, Figure 3).

## Discussion

Our systematic review suggests that *S. pneumoniae* is responsible for 19% of CAP in Indian patients >12 years of age. In a recently published study, Para et al evaluated the microbial aetiology of CAP in adult patients in a tertiary care hospital from North India. They observed that *S. pneumoniae* was the most common micro-organism accounting for nearly 31% of cases. Other organisms identified were *L. pneumophila* (17.5%), influenza viruses (15.4%), and *M. pneumoniae* (7.2%), with 4% of patients having multiple etiologies.^27^ Kumar et al recently conducted a study from South India to assess CAP among children between 2 months and 16 years of age. They observed *M. pneumoniae* as the most common pathogen (20% cases), followed by respiratory syncytial virus isolated in about 11% cases.^28^

Historically, common laboratory tests for pneumonia have included leukocyte count, sputum Gram stain, two sets of blood cultures, and urine antigens. Although previous administration of antibiotics may contribute to false-negative cultures rates, culture positivity rate values for the microbiological diagnosis of *S. pneumoniae* CAP by sputum culture may be confounded by upper airway contamination leading to false-positive culture rates.^29^ Furthermore, while isolation of *S. pneumoniae* from the sputum may represent colonization and overestimate its role in CAP, the prevalance of *S. pneumoniae* as a cause of CAP is underestimated due to lack of sensitivity of isolation technique from the blood. The urinary antigen method may enhance the sensitivity to detect *S. pneumoniae*; however, only two studies included in the present review performed urinary antigen testing, which was to detect *L. pneumophila*.^29^ Our results suggest that approximately one-fifth of adult Indian CAP patients had *S. pneumonia* identified as an aetiological agent. The other predominant aetiological agents reported in adult Indian CAP patients were *K. pneumoniae* (10.5%) and *M. pneumoniae* (15.4%).

Our results are in corroboration with the studies conducted in other parts of the world. For example, a Spanish study conducted on 109 CAP patients, found *S. pneumoniae* to be responsible for 25% of all cases.^30^ In the United Kingdom, the Research Committee of the British Thoracic Society and the Public Health Laboratory Service conducted a study to determine the aetiology of CAP in adult British patients. *S. pneumoniae* was identified as the foremost aetiological agent.^31^ Similar results were reported in adult CAP patients in other studies.^32,33^ The results from the studies indicate the importance and contribution of *S. pneumoniae* in the burden of CAP across the globe over long periods.

CAP is a major cause of adult mortality across Asia.^34^ Similar mortality patterns exist in developed western countries as well; for example, CAP is the sixth leading cause of death in the USA.^35^ People in the older age group have increased mortality due to CAP as compared with the younger age group, with Chronic Obstructive Pulmonary Disease (COPD) being a common predisposing condition for CAP in the elderly.^34,35^

These deaths can be prevented by promoting vaccination against CAP in susceptible adults, the coverage of which is still far from being adequate in India.^36^ Apart from focusing on optimal use of antibiotics in this antibiotic-resistance era, vaccination against the common causative organism may be of substantial preventive benefit against adult CAP in India. Furthermore, it may also reduce the economic burden due to CAP.^37^

There are two types of pneumococcal vaccines currently used globally – conjugate vaccines that contain 10 (PCV-10) or 13 (PCV-13) pneumococcal serotypes, and the plain polysaccharide vaccine that contains 23 pneumococcal serotypes (PPV23).^38^ Polysaccharide pneumococcal vaccine (PPV) was first introduced in 1983.^38^ The dawn of the 21st century saw the introduction of pneumococcal conjugate vaccine (PCV): heptavalent followed by 10-valent and 13-valent.^39^ In contrast to the plain polysaccharide vaccine, the conjugate vaccines induce T-cell dependent immune response. With respect to elderly adults, CAPITA, a randomized, double-blind, placebo-controlled trial, involving nearly 85 000 adults sought to establish the safety and efficacy of PCV13. Vaccine-serotype-specific CAP (diagnosed by either blood culture or a serotype-specific urine antigen detection assay) occurred in 49 participants in the PCV13 group as compared with 90 in the placebo group (vaccine efficacy, 45.6%; 95.2% CI, 21.8-62.5).^40^

The strength of this meta-analysis is that the included studies represent each of the regions of India. In this respect, an important consideration was the heterogeneity of included Indian studies. The random effects model found a varied distribution pattern. Our qualitative analysis also revealed similar results, but the source of heterogeneity could not be identified among the studies. The sensitivity analysis on 11 studies after excluding the study with the largest sample size led to comparable observations, suggesting that pooling these studies despite the difference in methodology was reasonable for this meta-analysis.

Nonetheless, a major limitation of this analysis was that the inclusion of all the existing eligible participants having different comorbidities could not be guaranteed. The other limitation was the reliance on sputum culture to make the microbiological diagnosis, in spite of the fact that the 17 studies included in this systematic review and meta-analysis were done in tertiary care hospitals in the urban area. Although most reported diagnosis based on clinical signs and symptoms along with microbiological tests (sputum culture and blood culture), 6 of 17 reported diagnoses were based on sputum culture and leukocyte count.

In addition, with the criteria established for this analysis, studies on viral pneumonia, or a comparison of viral with bacterial aetiology, were not included. Also, unpublished data such as conference abstracts or papers presented at scientific symposia were not included in the current study. Furthermore, no Indian study included in this study had reported multiple or mixed infections.

Future research should focus on larger epidemiological studies to identify aetiological organisms of CAP. This will help to establish a precise estimate and reliable association between the aetiological agents and CAP.

## Conclusions

This systematic review and meta-analysis identified the aetiological agents of bacterial CAP from published studies in the adolescent and adult Indian population, finding the predominant causes to be *S. pneumonia* (19%), *M. pneumoniae* (15.4%), and *K. pneumoniae* (10.5%).

## Supplemental Material

Supplemental – Supplemental material for Streptococcus pneumoniae as a Cause of Community-Acquired Pneumonia in Indian Adolescents and Adults: A Systematic Review and Meta-Analysis Click here for additional data file. Supplemental material, Supplemental for Streptococcus pneumoniae as a Cause of Community-Acquired Pneumonia in Indian Adolescents and Adults: A Systematic Review and Meta-Analysis by Canna J. Ghia, Raja Dhar, Parvaiz A Koul, Gautam Rambhad and Mark A Fletcher in Clinical Medicine Insights: Circulatory, Respiratory and Pulmonary Medicine
