# Supplementary material for: Streptococcus pneumoniae as a Cause of Community-Acquired Pneumonia in Indian Adolescents and Adults: A Systematic Review and Meta-Analysis
Source: Clin Med Insights Circ Respir Pulm Med. 2019 Jul 31;13:1179548419862790. doi: 10.1177/1179548419862790 (PMC6669839; doi:10.1177/1179548419862790)
Supplement: Supplemental – Supplemental material for Streptococcus pneumoniae as a Cause of Community-Acquired Pneumonia in Indian Adolescents and Adults: A Systematic Review and Meta-Analysis [file Supplemental.pdf]

Supplementary Tables

Table 1: Details of Risk of Bias Analysis

Criteria
1, Was the sample representative of the target population?
2, Were study participants recruited in an appropriate way?
3, Was the sample size adequate?
4, Were the study subjects and the setting described in detail?
5, Was the data analysis conducted with sufficient coverage of the identified sample?
6, Were objective, standard criteria used for the measurement of the condition?
7, Was the condition measured reliably?
8, Was there appropriate statistical analysis?
9, Are all important confounding factors/subgroups/differences identified and accounted for?
10, Were subpopulations identified using objective criteria?

**Publication**	**1**	**2**	**3**	**4**	**5**	**6**	**7**	**8**	**9**	**10**
Acharya et al., 2014	yes	unclear	yes	yes	yes	yes	yes	yes	yes	NA
Anbumani et al., 2010	unclear	unclear	no	yes	NA	yes	yes	NA	no	NA
Angrup, et al., 2016	yes	unclear	no	yes	yes	yes	yes	yes	yes	NA
Bansal, et al., 2004	yes	no	no	no	yes	yes	yes	yes	yes	NA
Dey, et al., 2000	yes	unclear	no	yes	yes	yes	yes	yes	yes	NA
Dey, et al., 1997	yes	unclear	no	yes	yes	yes	yes	yes	yes	yes
Dharmadhikari, et al., 2013	yes	unclear	no	no	unclear	yes	yes	yes	no	NA
Dorairaj, et al., 2015	yes	unclear	no	yes	yes	no	yes	no	no	NA
Jain, et al., 2014	yes	unclear	no	yes	yes	no	yes	yes	no	yes
Kejriwal, et al., 2015	yes	unclear	no	yes	yes	no	yes	no	no	NA
Khadanga, et al., 2014	yes	unclear	no	no	yes	yes	yes	no	no	NA
Menon, et al., 2013	yes	unclear	no	yes	yes	no	yes	NA	no	NA
Mythri, et al., 2013	yes	unclear	no	yes	yes	yes	yes	yes	no	NA
Ravindranath, et al., 2016	yes	unclear	no	yes	yes	no	yes	no	no	yes
Shah, et al., 2010	yes	unclear	no	yes	no	no	yes	no	yes	NA
Shrikhande, et al., 2015	yes	unclear	no	yes	no	no	yes	no	no	NA
Sreekanth and Reddy, 2015	yes	unclear	no	yes	no	no	yes	yes	no	yes

For Peer Review

Table 2: Etiology Data of Studies for All Organisms

Etiological Agent (EA)	No. of subjects with EA	Total No. of subjects with confirmed culture report	Total No. of subjects with CAP	Percentage of Occurrences	Reference First Author (Year)
S. pneumoniae	19	65	104	18.3%	Dharmadhikari *et al.*, 2013
	10	38	100	10.0%	Mythri *et al.*,2013
	10	29	50	20.0%	Sreekanth and Reddy, 2015
	19	53	70	27.1%	Bansal *et al.*, 2003
	6	11	72	8.3%	Dey *et al.*, 1997
	68	149	464	14.7%	Khadanga *et al.*, 2014
	1	29	100	1.0%	Shah *et al.*, 2010
	20	55	120	16.7%	Jain *et al.*, 2014
	34	60	60	56.7%	Kejriwal *et al.*, 2015
	11	26	50	22.0%	Shrikhande *et al.*, 2015
	12	39	100	12.0%	Acharya *et al.*, 2014
	47	145	145	32.4%	Menon *et al.*, 2013
**Total**	**257**	**699**	**1435**	**Range= 1.0–56.7%**	**19.0%**
K. pneumoniae	17	65	104	16.34%	Dharmadhikari *et al.*, 2013
	21	38	100	21.0%	Mythri *et al.*,2013
	12	29	50	24.0%	Sreekanth and Reddy, 2015
	12	53	70	17.14%	Bansal *et al.*, 2003
	3	11	72	4.16%	Dey *et al.*,1997
	29	149	464	6.25%	Khadanga *et al.*, 2014
	3	29	100	3.0%	Shah *et al.*, 2010
	16	55	120	13.33%	Jain *et al.*,

For Peer Review

Etiological Agent (EA)	No. of subjects with EA	Total No. of subjects with confirmed culture report	Total No. of subjects with CAP	Percentage of Occurrences	Reference First Author (Year)
					2014
	1	60	60	1.6%	Kejriwal *et al.*, 2015
	3	26	50	6.0%	Shrikhande *et al.*, 2015
	5	39	100	5.0%	Acharya *et al.*, 2014
	29	145	145	20.0%	Menon *et al.*, 2013
**Total**	**151**	**699**	**1435**	**Range= 1.6–24.0%**	**10.52%**
M. pneumoniae	8	53	70	1.14%	Bansal *et al.*, 2003
	22	62	62	35.48%	Dey *et al.*, 2000
	7	13	107	6.54%	Dorairaj *et al.*, 2015
**Total**	**37**	**128**	**239**	**Range=1.14–35.48%**	**15.48%**
P. aeruginosa	8	65	104	7.69%	Dharmadhikari *et al.*, 2013
	4	38	100	4.0%	Mythri *et al.*,2013
	2	29	50	4.0%	Sreekanth and Reddy, 2015
	2	53	70	2.85%	Bansal *et al.*, 2003
	1	11	72	1.38%	Dey *et al.*, 1997
	31	149	464	6.68%	Khadanga *et al.*, 2014
	10	29	100	10.0%	Shah *et al.*, 2010
	1	55	120	0.83%	Jain *et al.*, 2014
	7	60	60	11.6%	Kejriwal *et al.*, 2015
	4	26	50	8.0%	Shrikhande *et al.*, 2015
	6	39	100	6.0%	Acharya *et al.*, 2014
	13	145	145	8.96%	Menon *et al.*, 2013
**Total**	**89**	**699**	**1435**	**Range= 0.83–11.6%**	**6.20%**
S. aureus	13	65	104	12.5%	Dharmadhikari *et al.*, 2013

For Peer Review

Etiological Agent (EA)	No. of subjects with EA	Total No. of subjects with confirmed culture report	Total No. of subjects with CAP	Percentage of Occurrences	Reference First Author (Year)
	1	38	100	1.0%	Mythri *et al.*,2013
	1	29	50	2.0%	Sreekanth and Reddy, 2015
	9	53	70	12.85%	Bansal *et al.*, 2003
	12	149	464	2.58%	Khadanga *et al.*, 2014
	7	29	100	7.0%	Shah *et al.*, 2010
	15	27	150	10.0%	Ravidranath and Raju, 2016
	11	55	120	9.16%	Jain *et al.*, 2014
	2	60	60	3.33%	Kejriwal *et al.*, 2015
	5	26	50	10.0%	Shrikhande *et al.*, 2015
	3	39	100	3.0%	Acharya *et al.*, 2014
**Total**	**79**	**570**	**1468**	**Range= 1.0–12.8%**	**5.38%**
Acinetobacter	3	65	104	2.88%	Dharmadhikari *et al.*, 2013
	2	29	50	4.0%	Sreekanth and Reddy, 2015
	1	53	70	1.4%	Bansal *et al.*, 2003
	1	29	100	1.0%	Shah *et al.*, 2010
	1	55	120	0.83%	Jain *et al.*, 2014
	3	60	60	5.0%	Kejriwal *et al.*, 2015
	3	39	100	3.0%	Acharya *et al.*, 2014
**Total**	**14**	**330**	**604**	**Range= 0.83–5.0%**	**2.31%**
Enterobacter	2	29	50	4.0%	Sreekanth and Reddy, 2015
	1	53	70	1.42%	Bansal *et al.*, 2003
	1	55	120	0.83%	Jain *et al.*, 2014

For Peer Review

Etiological Agent (EA)	No. of subjects with EA	Total No. of subjects with confirmed culture report	Total No. of subjects with CAP	Percentage of Occurrences	Reference First Author (Year)
	1	39	100	1.0%	Acharya *et al.*, 2014
**Total**	**5**	**176**	**340**	**Range= 0.83–4.0%**	**1.47%**
E. coli	4	65	104	3.84%	Dharmadhikari *et al.*, 2013
	1	38	100	1.0%	Mythri *et al.*,2013
	2	29	50	4.0%	Sreekanth and Reddy, 2015
	6	53	70	8.57%	Bansal *et al.*, 2003
	6	29	100	6.0%	Shah *et al.*, 2010
	1	55	120	0.83%	Jain *et al.*, 2014
	3	60	60	5.0%	Kejriwal *et al.*, 2015
	3	26	50	6.0%	Shrikhande *et al.*, 2015
	3	39	100	3.0%	Acharya *et al.*, 2014
	9	145	145	6.20%	Menon *et al.*, 2013
**Total**	**38**	**539**	**899**	**Range= 0.83–8.57%**	**4.22%**
L. pneumophila	12	470	470	2.5%	Anbumani *et al.*, 2010
	32	134	134	23.88%	Angrup *et al.*, 2016
**Total**	**44**	**604**	**604**	**Range= 2.5–23.8%**	**7.28%**
